# Peptide Helix-Y^12^ as Potential Effector for Peroxisome Proliferator-Activated Receptors

**DOI:** 10.1155/2023/8047378

**Published:** 2023-04-15

**Authors:** Mauricio Carrillo-Tripp, Yair Reyes, Blanca Delgado-Coello, Jaime Mas-Oliva, Roxana Gutiérrez-Vidal

**Affiliations:** ^1^Biomolecular Diversity Laboratory, Centro de Investigación y de Estudios Avanzados del Instituto Politécnico Nacional, Unidad Monterrey, Vía del Conocimiento 201, PIIT, C.P. 66600, Apodaca, Nuevo León, Mexico; ^2^Metabolic Diseases Laboratory, Centro de Investigación y de Estudios Avanzados del Instituto Politécnico Nacional, Unidad Monterrey, Vía del Conocimiento 201, PIIT, C.P. 66600, Apodaca, Nuevo León, Mexico; ^3^Universidad Politécnica de Puebla, Tercer Carril del Ejido, Serrano s/n, Cuanalá, C.P. 7264, Puebla, Mexico; ^4^Instituto de Fisiología Celular, Universidad Nacional Autónoma de México, C.P. 04510, CDMX, Mexico; ^5^Programa de Investigadoras e Investigadores por México, Conacyt, CDMX, Mexico

## Abstract

Peroxisome proliferator-activated receptors (PPARs) are nuclear receptors involved in the regulation of lipids and glucose metabolism, and immune response. Therefore, they have been considered pharmacological targets for treating metabolic diseases, such as dyslipidemia, atherosclerosis, and non-alcoholic fatty liver disease. However, the available synthetic ligands of PPARs have mild to significant side effects, generating the necessity to identify new molecules that are selective PPAR ligands with specific biological responses. This study aimed to evaluate some components of the atheroprotective and hepatoprotective HB-ATV-8 nanoparticles [the amphipathic peptide Helix-Y^12^, thermozeaxanthin, thermozeaxanthin-13, thermozeaxanthin-15, and a set of glycolipids], as possible ligands of PPARs through blind molecular docking. According to the change in free energy upon protein–ligand binding, ∆*G*_b_, thermozeaxanthins show a more favorable interaction with PPARs, followed by Helix-Y^12^. Moreover, Helix-Y^12^ interacts with most parts of the Y-shaped ligand-binding domain (LBD), surrounding helix 3 of PPARs, and reaching helix 12 of PPAR*α* and PPAR*γ*. As previously reported for other ligands, Tyr314 and Tyr464 of PPAR*α* interact with Helix-Y^12^ through hydrogen bonds. Several PPAR*α*'s amino acids are involved in the ligand binding by hydrophobic interactions. Furthermore, we identified additional PPARs' amino acids interacting with Helix-Y^12^ through hydrogen bonds still not reported for known ligands. Our results show that, from the studied ligand set, the Helix-Y^12^ peptide and Tzeaxs have the most significant probability of binding to the PPARs' LBD, suggesting novel ligands for PPARs.

## 1. Introduction

Peroxisome proliferator-activated receptors (PPARs) are ligand-activated transcription factors that regulate genes involved in several biological processes, mainly glucose and lipid metabolism, as well as immune response. This family of nuclear receptors includes the isoforms PPAR*α*, PPAR*β*/*δ*, and PPAR*γ*, each encoded by a different gene located on separate chromosomes, but with a high degree of sequence and structural homology [1].

Tissue distribution patterns and expression levels are the main differences between the three isoforms, displaying regulatory activities and modulating specific responses [2]. PPAR*α* is expressed predominantly in skeletal muscle, heart, liver, and brown adipose tissue, all of them are high-energy-requiring tissues. There is also an important expression of PPAR*α* in cells composing the vasculature, such as endothelial cells, smooth muscle cells, and monocytes/macrophages [3–6]. PPAR*γ* is highly expressed in adipose tissue and macrophages, and at much lower levels in the liver and muscle. This isoform has been considered as a master regulator of adipogenesis, lipid storage, insulin sensitivity, and immune regulation. Moreover, the expression of PPAR*α* and PPAR*γ* in the subendothelial region and the lipid core of atherosclerotic lesions, where they colocalize with specific markers of macrophages, smooth muscle cells, and foam cells, has been described [7]. PPAR*β*/*δ*, the third family member, shows expression in a variety of tissues, such as heart, vascular smooth muscle cells, adipose tissue, brain, intestine, muscle, spleen, lung, and adrenal glands [8].

The tertiary structure of the PPARs has a DNA binding domain in the N-terminus and a ligand-binding domain (LBD) in the C-terminus. The LDB is a Y-shaped pocket composed of 13 *α*-helices and 4 *β*-sheets. This pocket contains the activation function (AF-2) region that serves as a binding site for coregulator proteins [9], and the helix H12 is considered as the activating site [10]. The dynamic conformation of LBD is stabilized with PPAR ligand binding, promoting interactions with coregulator proteins, in turn remodeling the chromatin, facilitating polymerase binding, and expression of target genes [9]. Although, ligand-dependent transactivation of PPARs is one of their mechanisms; they also present ligand-independent repression and ligand-dependent transrepression [11, 12].

The main ligands for PPARs are endogenous lipid-soluble fatty acids and their derivatives, such as eicosanoids, prostaglandins, and leukotriene B4 [13]. Examples of these fatty acids that interact in LBD are conjugated linoleic acid, 9-(s)-hydroxyoctadecadienoic, and 15-deoxydelta12,14-prostaglandin J12, which are known endogenous and dietary agonists [14]. Ligands can also be synthetic, such as Wy-14643 and fibrates (specific PPAR*α* activators) [13]. The latter have proven to increase triglyceride catabolism through overexpression of lipoprotein lipase and reduce the secretion of chylomicrons in enterocytes. They also increase circulating levels of atheroprotective high-density lipoprotein cholesterol [15], and improve the overall atherogenic plasma lipid profile. GW501516 is a highly-selective PPAR*β*/*δ* ligand, and thiazolidinedione (TZD) derivatives (troglitazone, pioglitazone, GW1929, and GW2090) are specific PPAR*γ* activators [13]. In the vasculature, PPARs agonists have a role in vascular metabolism involved in recruitment and adhesion of inflammatory cells. They also induce nitric oxide synthase expression by increasing nitric oxide bioavailability, suggesting vasculoprotective effects [16, 17]. Furthermore, PPAR*α* increases the expression of I*κ*B, an inhibitor of the pro-inflammatory transcription factor nuclear factor *κ* beta (NF-*κ*B). It also inhibits many inflammatory genes, such as NF-*κ*B, activator protein 1, and nuclear factor of activated T cells by transrepression. PPAR*α* activation reduces the production of proinflammatory cytokines, such as interleukin-1 and interleukin-6, thus preventing the expression of adhesion molecules, such as vascular cell adhesion molecule-1 and intracellular cell adhesion molecule-1 [18–20]. In mice, PPAR*α* activation using fibrates proved to be protective for acute liver injury [21]. TZDs are potent PPAR*γ* agonists that increase insulin sensitivity and induce browning of white fat [22]. Furthermore, activation of PPAR*γ* by a TZD (pioglitazone) has vascular protective action explained through ligand-dependent formation of the complex of PPAR*γ–*high mobility group A1-SUMO E2 ligase Ubc9 that promote PPAR*γ* SUMOylation, necessary for the metalloproteinase-9 transrepression, a key mediator of vascular injury [12]. However, TZDs are associated with several side effects, including gain in body weight and visceral obesity. Selective PPAR*γ* modulators (amorfrutins) have been shown to improve insulin sensitivity, dyslipidemia [23], liver steatosis [24], and atherosclerosis [25], without increasing body weight. It has been shown that a partial activator of PPAR*γ*, telmisaten, in combination with activating PPAR*α* in the liver, could alleviate hepatic steatosis in mice that were fed a high-fat diet [26]. According to the literature, PPAR agonists are suitable drug targets for treating highly prevalent metabolic diseases, such as type 2 diabetes, atherosclerosis, and non-alcoholic fatty liver disease (NAFLD). However, given the low efficacy and side effects of current PPAR agonists, there is a need for the development or identification of new molecules that are safe and selective PPAR ligands to obtain a more specific agonist activity to promote selected biological responses [27, 28].

We have previously shown that HB-ATV-8 lipid nanoparticles have a protective effect against atherosclerotic lesions and NAFLD induced by a high-fat diet in pigs and rabbits [29, 30]. The nanoparticles are mainly composed of membrane lipids of *Thermus aquaticus* and an amphipathic peptide (Cys+ the last 11 residues of the cholesteryl-ester transfer protein, called Helix-Y^12^). The HB-ATV-8 nanoparticles induce mild cholesteryl-ester transfer protein antibodies production, explaining in part the observed effect. However, the protective effect is present even without antibody production [29]. Hence, we suggest that peptide Helix-Y^12^ and the lipid components of the nanoparticles could participate directly in the biological effect. The membrane of *T. aquaticus* has abundant carotenoids, such as thermozeaxanthins (Tzeaxs), glycolipids, and glycerophospholipids. Carotenoids have antioxidant properties, also reported as bioactive lipids that bind to receptors and transcription factors modifying several related-pathways, lipids, and inflammation [31–35].

In this study, we evaluate a set of molecules found in the HB-ATV-8 formulation, namely, the Helix-Y^12^ peptide and main lipids from the membrane of *T. aquaticus*, to identify potential effectors of PPAR*α*, PPAR*β*/*δ*, and PPAR*γ* based on the change in free energy after protein–ligand binding and the types of interactions found through blind molecular coupling. Our results show that the peptide Helix-Y^12^ and the Tzeaxs establish favorable interactions with the LBD of the three PPARs. However, the peptide establishes more hydrogen bonds than some Tzeaxs, forming a clamp inside the PPARs LBD reaching H3 and H12 helices. These findings shed light into the mechanisms of action of the nanoparticles that protect against atherosclerotic and hepatic lesions induced by a high-fat diet.

## 2. Methods

### 2.1. Target and Ligand Molecular Modeling

**Figure 1:**
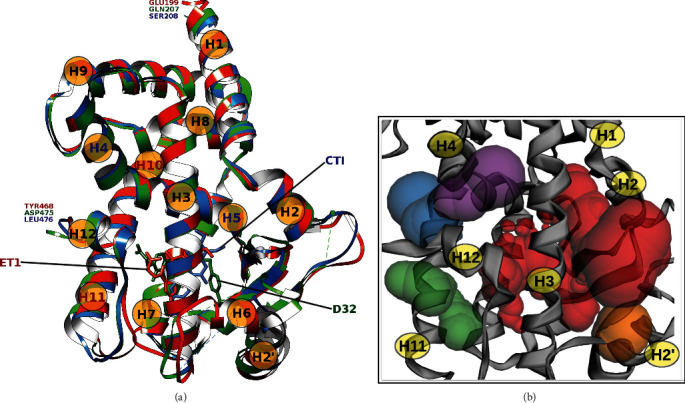
Molecular structure of protein targets: (a) co-crystallized in complex with a corresponding active molecule in the LBD (holo state) PPAR*α*-ET1 (PDBID: 3ET1, in red), PPAR*β*/*δ*-D32 (PDBID: 3GZ9, in green), and PPAR*γ*-CTI (PDBID: 4Y29, in blue). (b) Top 5 cavities, ranked by size, LBD in red 458 Å^3^, green 63 Å^3^, blue 52 Å^3^, purple 42 Å^3^, and orange 1 Å^3^ (volumes calculated with the CASTp 3.0 [76] on 3GZ9). Helix pairs H4–H5 and H10–H11 are continuous secondary structures.

**Figure 2:**
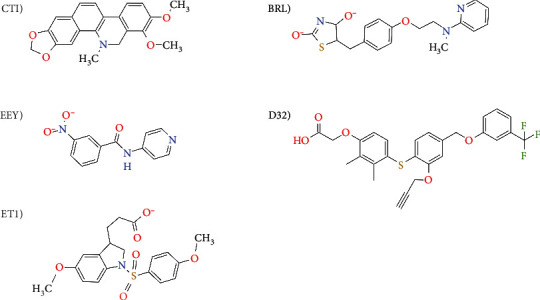
Chemical structure of the active molecules in the positive control group used in the molecular docking: ET1 for PPAR*α*, D32 for PPAR*β*/*δ*, and CTI, EEY, and BRL for PPAR*γ*.

The three-dimensional coordinates of the crystallographic structure of PPAR*α* (PDBID: 3ET1), PPAR*β*/*δ* (PDBID: 3GZ9), and PPAR*γ* (PDBID: 4Y29) were obtained from the RCSB PDB database [36], shown in Figure 1. Structural data curation involved deleting solvent and non-complexed ions, keeping the highest occupancy atom locations, replacing incomplete side chains using the Dunbrack 2010 rotamers, and adding hydrogen atoms, using the Chimera's Dock Prep tools v 1.13.1 [37, 38]. We built a positive control group composed of the ligands found in the co-crystallization experiments of the target molecules to validate the blind molecular docking methodology is shown in Figure 2.

**Figure 3:**
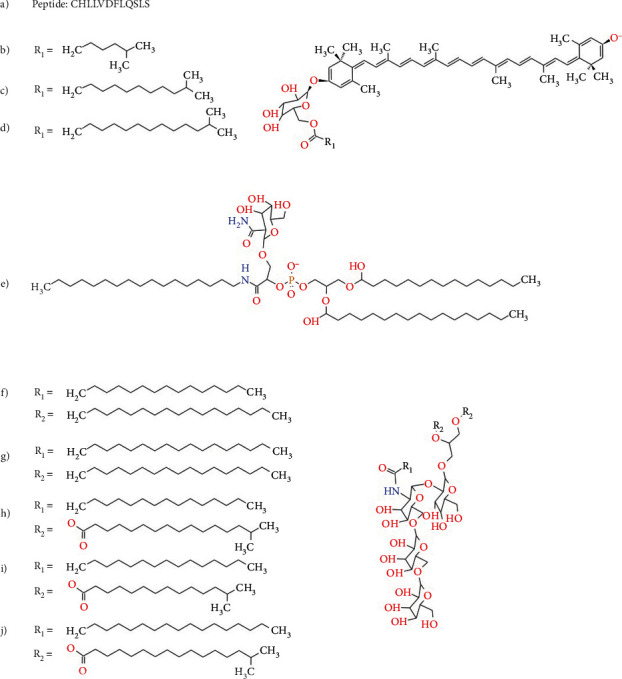
Chemical structure of the ligand molecules in the experimental group used for molecular docking: (a) Helix-Y^12^, (b) Tzeax, (c) Tzeax-13, (d) Tzeax-15, (e) gpl01, (f) gl01, (g) gl02, (h) gl03, (i) gl04, and (j) gl05.

Given the results reported in a previous study [39], we analyzed the interaction between PPARs and the 12 amino acids long amphipathic peptide, Helix-Y^12^. In addition, we included relevant components of HB-ATV-8 nanoparticles to build the experimental set of ligands is shown in Figure 3, because it is well known that PPARs are promiscuous toward lipids and their derivatives. Nanoparticles are composed of lipids from *T. aquaticus* cell's membrane, which contains phospholipids, glycolipids, and carotenoids. In this study, we selected the carotenoids Tzeax, thermozeaxanthin-13 (Tzeax-13), and thermozeaxanthin-15 (Tzeax-15) due to their high abundance in the *T. aquaticus* lipid membrane and their antioxidant properties, as well as some representative glycerophospholipids.

In all cases, the molecular structure of the ligands was built from scratch based on their chemical structure (Figures 2 and 3) using the Chimera v 1.13.1 [37]. After adding Gasteiger partial charges on all atoms based on the Amber 14SB force-field, a two-step minimization phase was done carrying out 3,000 steepest descent and 10,000 conjugate gradient steps with a 0.02 step size in both cases. The minimized structure was exported in MOL2 format and imported into the AutoDockTools v1.5.6 through the Ligand-input menu. All non-polar hydrogen atoms were merged with corresponding carbon atoms automatically, leaving only explicit polar hydrogen atoms, also detecting rotatable bonds (torsion degrees of freedom). At the end, the ligands molecular structures were exported in PDBQT format.

### 2.2. Protein–Ligand Blind Molecular Docking

After the target and ligand molecular modeling, a three-phase pipeline cycle was followed: target cavity detection, docking box optimization, and target–ligand docking. An in-house bash script was developed (https://github.com/tripplab/HTVS) [40] to implement and automatize the CB-Dock [41] and the AutoDock Vina [42] computational tools in a high-throughput fashion at a local high-performance computing multi-core server. Since CB-Dock is a cavity detection-guided protein–ligand blind docking, the curated target tertiary structure was analyzed to find all cavities in the protein's surface. This was accomplished by the CurPocket tool, which is based on a curvature-dependent surface-area model [43]. All detected cavities were ranked by surface area size, from largest to smallest. The ligand structure data and the location and size of the cavities found in the target's molecular surface were used to define a customized docking box, which was used as input parameters in the next phase. The AutoDock Vina has been shown to be a fast and accurate docking method based on a free energy scoring function that allows for efficient optimization and multithreading. It takes the PDBQT target and ligand structural data as inputs, as well as the docking box configuration optimized parameters.

For a full virtual screening, we provided the set of targets *T* and the set of ligands *L* (PDBQTs), the number of target cavities to sample *N*, and the number of independent rounds *K* to perform for each target–ligand pair. As a result, the pipeline outputs a numeric matrix with the change in binding free energy values ∆*G*_b_ for all target–ligand top conformations for each cavity into a database. The data was analyzed with the *heatmap.2(data.matrix(sheet))* R function. The rows and columns were independently scaled to have *mean* = 0 and *standard deviation* = 1 to generate two *Z*-score based heat-map representations of the data. Furthermore, two-dimensional diagrams from the predicted protein–ligand complexes were generated using the Ligplot [44] to identify the PPARs LBD amino acids involved in the favorable interaction with the corresponding ligand.

## 3. Results and Discussion

**Figure 4:**
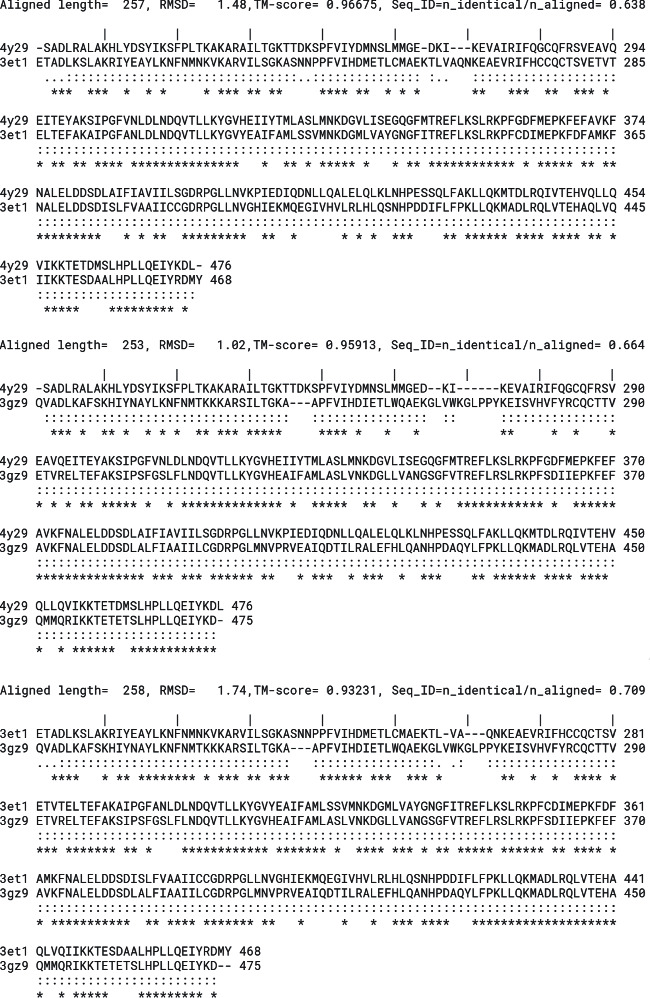
Structure-based sequence alignment of the three PPAR variants. Top to bottom: PPAR*γ* (PDBID 4y29) versus PPAR*α* (PDBID 3et1), PPAR*γ* (PDBID 4y29) versus PPAR*β*/*δ* (PDBID 3gz9), and PPAR*α* (PDBID 3et1) versus PPAR*β*/*δ* (PDBID 3gz9). For each pair of variants, aligned length, RMSD, TM-score, and sequence identity were reported using the TM-align algorithm. For each aligned residue pair, “:” denotes distance <5 : 0 Å, “.” denotes farther aligned residues, and “∗” denotes identical residues.

The structural superposition of the three LBD of the PPARs (Figure 1(a)) shows that the pair-wise C*α* Root Mean Square Deviation (RMSD) is below 2 Å and the TM-score is above 0.9, indication that they share the same protein fold. However, the sequence identity is relatively low (Figure 4), hence, we considered them as three independent targets for the molecular docking (Table 1).

For every target–ligand pair, we ran consecutive independent blind molecular docking cycles, each one sampling the top 5 cavities detected on the surface of the target (Figure 1(b)). We continued running cycles until we no longer found lower ∆*G*_b_ values, suggesting a sufficiently large sampling of the conformational space. We found that 20 independent cycles for each pair were enough. We then selected the conformation with the largest binding free-energy negative change at all cavities from all the independent cycles performed for each target–ligand complex. The generated data of all systems studied here follow the FAIR principles [46] and can be accessed and visualized at the MDdb Science Gateway at https://www.md-db.org with Study ID 690004.

### 3.1. PPARs Co-Crystallized Ligand Complexes Are Reproduced by the Blind Docking Protocol

**Figure 5:**
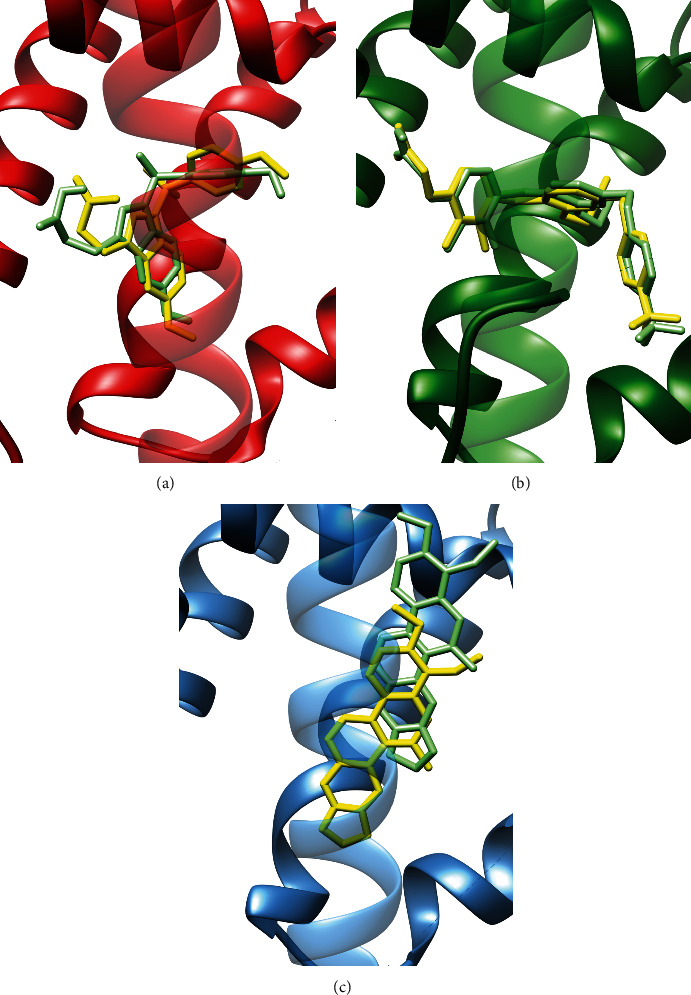
Results for the active molecules in the positive control group: (a) ET1 for PPAR*α* (in red), (b) D32 for PPAR*β*/*δ* (in green), and (c) CTI for PPAR*γ* (in blue). In all cases, co-crystalized ligand in yellow, blind docking prediction in green, and PPAR helix H3 transparent.

The ample blind conformational sampling correctly predicted the biologically active LBD cavity for all the co-crystallized ligands in the positive control group with the lowest ∆*G*_b_ values in the corresponding target. The superposition of the crystal structure and the docked conformation reveals that the ligands are predicted to bind to the same site and maintain a similar pose as the ligand in the crystal structure in all cases (Figure 5), namely, ET1 for PPAR*α* (RMSD = 5.016), D32 for PPAR*β*/*δ* (RMSD = 0.782), and CTI for PPAR*γ* (RMSD = 5.059). This evidence suggests that the general pipeline employed in this study is reliable and produces accurate results.

### 3.2. Global Analysis of the Ligand Set and PPARs

**Figure 6:**
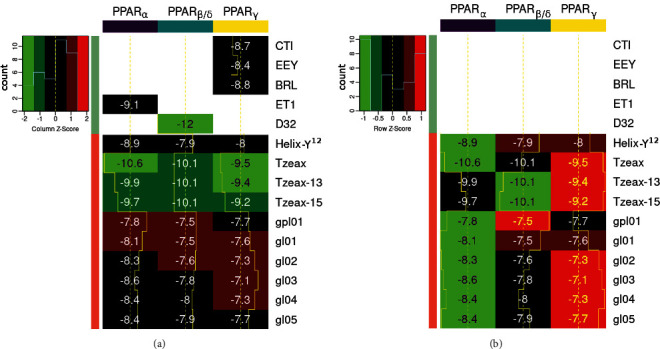
Target–ligand ∆*G*_b_ values (kcal/mol) at the biologically active cavity of PPAR*α* (purple), PPAR*β*/*δ* (blue), and PPAR*γ* (yellow). Statistical *Z*-score analysis heat-map (a) for the ligand set (columns) and (b) for the target set (rows).

The ∆*G*_b_ values for the blind docking results of the three isoforms of PPARs and some molecular components of the HB-ATV-8 nanoparticles (peptide Helix-Y^12^ and main lipids of the plasmatic membrane of *T. aquaticus*) are shown in Figure 6. Based on the statistical *Z*-score analysis done over the ligand set for each PPAR (columns), the Tzeax, Tzeax-13, and Tzeax-15 are the components of the HB-ATV-8 nanoparticles that have the most favorable thermodynamic interaction with all three isoforms of PPARs, followed by peptide Helix-Y^12^ (Figure 6(a)). However, the interaction of the peptide with PPAR LBD shows more hydrogen bonds than that of the Tzeaxs. This result suggests a specific interaction between the PPARs and the peptide than the Tzeaxs under physiological conditions.

When the statistical *Z*-score analysis was performed on the target set for each ligand (rows), we found PPAR*α* had the most favorable thermodynamic interaction with Helix-Y^12^ and Tzeax across the three isoforms. Meanwhile, PPAR*β*/*δ* had the most favorable thermodynamic interaction with Tzeax-13 and Tzeax-15 (Figure 6(b)). According to our analysis, the less favorable receptor for this set of ligands seems to be PPAR*γ*. In this sense, PPAR*α* LBD has an affinity for a broader range of saturated fatty acids than PPAR*γ* and PPAR*β*/*δ* [47], explained by having the most lipophilic ligand-binding pocket of the three isoforms [48].

### 3.3. Helix-Y^12^ Forms a Stabilizing Hydrogen Bond Network with PPARs

**Figure 7:**
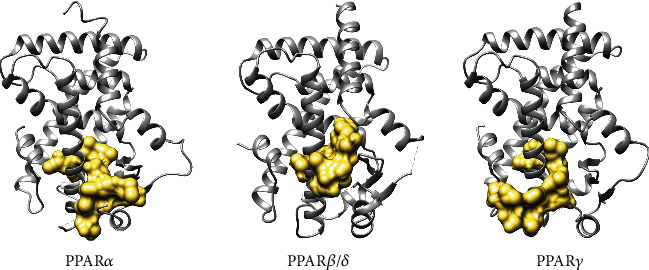
Ribbon diagrams of PPAR*α*, PPAR*β*/*δ*, and PPAR*γ* (grey) bound to surface diagram of peptide Helix-Y^12^ (gold).

Helix-Y^12^ is an amphipathic peptide that interacts with the whole Y-shaped ligand-binding pocket surface of the PPARs LBD, as shown in Figure 7. We found that a hydrogen bond network is formed between the PPARs and this peptide, although it involves different protein residues in each isoform.

**Figure 8:**
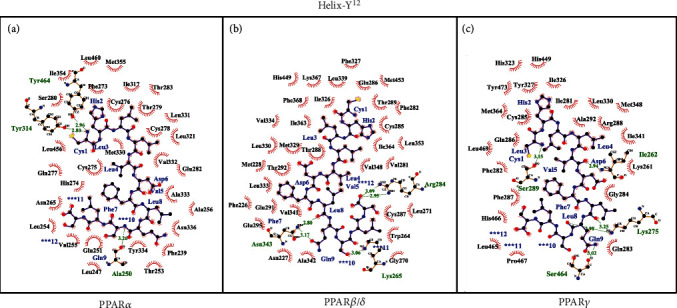
Interatomic interaction of peptide Helix-Y^12^ (label in blue) with (a) PPAR*α*, (b) PPAR*β*/*δ*, and (c) PPAR*γ*. Hydrogen bonds are indicated with green label and hydrophobic forces in black one.

Helix-Y^12^ makes three hydrogen bonds via Cys1 and Ser10 with residues Tyr314, Tyr464, and Ala250 of PPAR*α* LBD (Figure 8(a)). There are also hydrophobic interactions involving *β*-sheets (13%) and H3 (30%). It should be noted that Helix-Y^12^ binds to PPAR*α* LBD through hydrogen bonds in Tyr314 and Tyr464, which have previously been reported to interact with several PPAR*α* ligands. PPAR*α* crystallographic studies indicated that the interaction of ligands through Tyr464 and Tyr314 stabilizes the AF-2 helix, promoting the recruitment of coactivators [47, 49, 50]. Endogenous ligands (stearic and palmitic acid) and synthetic ligands (pemafibrate, saroglitazar, and GW7647) interact primarily with hydrophobic residues. Their carboxylic acid establishes a network of hydrogen bonds by common residues Tyr314, His440, Tyr 464, and Ser280. Synthetic ligands also interact through hydrogen bonds, involving other residues, such as Tyr334, Lys257, Cys275, Thr279, and Glu282 (ciprofibrate) and Ala333, Glu251, Leu331, Cys275, and Thr279 (pemafibrate) [50, 51]. Furthermore, several residues of PPAR*α* that participate in the hydrophobic effect with the peptide correspond to those that interact with fatty acids (stearic and palmitic acid) and fibrates derivatives (pemafibrate and ciprofibrate). Isothermal titration calorimetry and fragment molecular orbital studies pointed out that the accumulation of weak hydrophobic contacts is important for the binding affinity of pemafibrate to PPAR*α* LBD, as they confer a higher affinity for it compared with fenofibrate [51].

For PPAR*β*/*δ* LBD, Helix-Y^12^ also covers the entire Y-shaped ligand-binding pocket, but its contact with Arm X is minimal (Figure 7). Asn343, Lys265, and Arg284 interact via hydrogen bonds with Helix-Y^12^ residues Leu8, Gln9, Ser10, and Ser12 (Figure 8(b)). Hydrophobic contacts with H3 (29%), H5 (14%), loop H2′–H3 (11%), and H7 (11%) were also observed.

Finally, we show in Figure 7 that Helix-Y^12^ forms a clamp around H3 and reaches up to helix H12 of PPAR*γ*. In this complex, the peptide is in Arm I, center, and partially in Arm III, leaving a binding-ligand pocket through Arms II and X. The interatomic analysis shows that Ile262, Lys275, Ser289, and Ser464 of PPAR*γ* LBD interact with the peptide residues Cys1, Val5, Leu8, and Gln9 through hydrogen bonds (Figure 8(c)). In addition to these dipole–dipole attractive interactions, there are also hydrophobic contacts mainly with H3 (37%), H5 (15%), loop H11–H12 (11%), and H12 (11%). Of the amino acid residues that establish hydrogen bonds between Helix-Y^12^ and PPAR*γ*, Ser289 has been reported in full agonists, such as rosiglitazone and MLR-20. The rest are in a region essential for LBD organization, such as the loops H2′–H3 and H11–H12.

Table 2 summarizes relevant characteristics observed in the interaction of Helix-Y^12^ and PPARs. Although Helix-Y^12^ does not show the highest ∆*G*_b_ values, it established interactions with amino acids reported from the Y-shaped ligand-binding pocket that happen with other ligands. Most of them are hydrophobic but some can make hydrogen bonds, related to the specificity for PPARs [50]. Helix-Y^12^ surrounds helix *Η*3 of the PPAR LBD to a lesser or greater extent, occupying all three arms. In vitro studies have shown that peptide Helix-Y^12^ has biological effects on hepatocytes (in preparation). Altogether, these findings suggest that the peptide could be an exciting ligand that needs further studies to validate its interactions and a possible biological effect.

### 3.4. Tzeaxs Have the most Stable Interaction with PPARs

**Figure 9:**
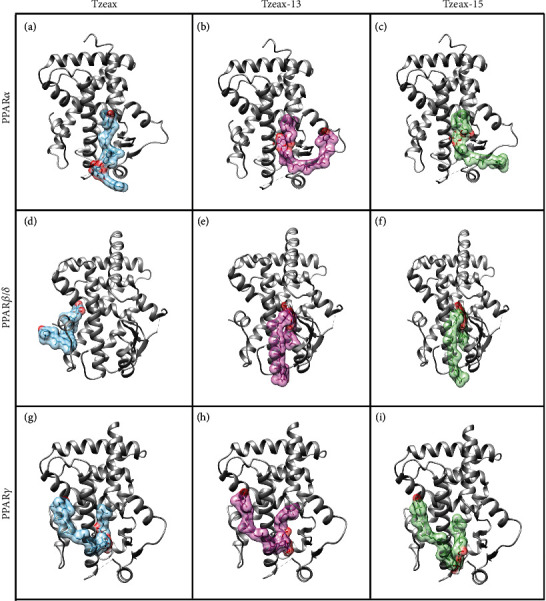
Ribbon diagrams of PPAR*α*, PPAR*β*/*δ*, and PPAR*γ* (grey) bound to surface/ribbon diagrams of (a, d, and g) Tzeax (blue), (b, e, and h) Tzeax-13 (pink), and (c, f, and i) Tzeax-15 (green).

The interaction of PPARs LBD with Tzeax, Tzeax-13, and Tzeax-15 is shown in Figure 9. As previously mentioned, Tzeax, Tzeax-13, and Tzeax-15 have the most favorable thermodynamic change in free energy of binding with the three isoforms of PPARs from all molecules tested.

According to the ∆*G*_b_ values for the blind docking results, Tzeax shows a more favorable interaction with PPAR*α* and PPAR*γ* than the ligands employed in the co-crystallization with the protein, ET1 and CTI, respectively. This is not the case for PPAR*β*/*δ*, where ligand D32 stands out over the entire set of components of HB-ATV-8.

Another opposite trend is also apparent between PPAR*α* and PPAR*γ* with PPAR*β*/*δ*. Decreasing the aliphatic chain length of the Tzeaxs improves the interaction stability for the former, whereas increasing the length improves the interaction stability for the later. All other ligands are close to or significantly less negative than the set's ∆*G*_b_ mean value.

### 3.5. Tzeaxs Interact with the PPARs Mainly by Hydrophobic Contacts

**Figure 10:**
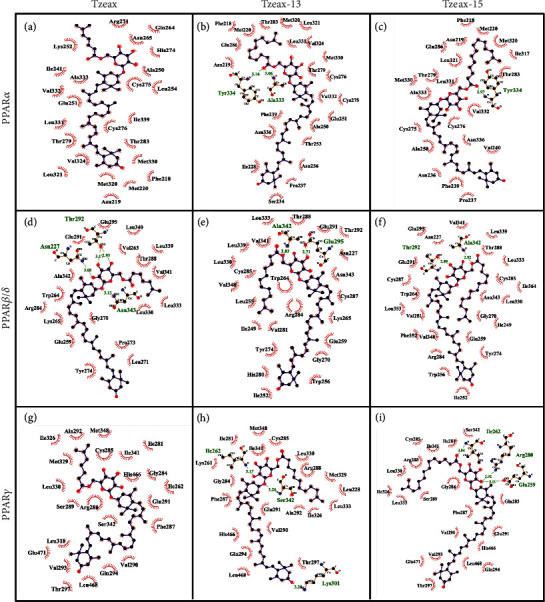
Interatomic interaction of thermozeaxanthins and PPAR-LBDs. Interatomic interaction of Tzeax with (a) PPAR*α*, (d) PPAR*β/δ*, and (g) PPAR*γ*, Tzeax-13 with (b) PPAR*α*, (e) PPAR*β/δ*, and (h) PPAR*γ*, and Tzeax-15 with (c) PPAR*α*, (f) PPAR*β/δ*, and (i) PPAR*γ*. All plots were generated using LigPlot. Labels in green correspond to residues involved in hydrogen bonds, and black one to those that interact by hydrophobic forces.

As previously mentioned, we found that Tzeax, Tzeax-13, and Tzeax-15 established contacts with the LBD PPARs, except for Tzeax and PPAR*β*/*δ* (Figure 9). For the Tzeax, the free polar ring of the zeaxanthin portion reaches Arm III of the ligand-binding pocket of PPAR*α*, and the rest of the molecule passes through Arms II and X, leaving the fatty acid fraction on the outside (Figure 9(a)). However, all the contacts are due to the hydrophobic effect. They are mainly found in H3 (25%), loops (25%), and *β*-sheets (17%; Figure 10(a)). In contrast, the ester bond of Tzeax-13 located in the Arm II of the ligand-binding pocket of PPAR*α* establishes hydrogen-bonds with the amide group of Tyr334 and Ala333 (Figures 9(b) and 10(b)). In addition to hydrogen bonds, there are hydrophobic contacts with H2′ (13%), H3 (21%), H5 (13%), *β*-sheets (17%), and loop H1–H2 (13%). On the other hand, Figure 10(c) shows how Tzeax-15 forms a direct hydrogen bond with Tyr334 of PPAR*α* LBD, and hydrophobic contacts with H3 (23%), H5 (14%), and *β*-sheets (23%). Both Tzeax-13 and Tzeax-15 reach Arm III of the ligand-binding pocket of PPAR*α* with the fatty acid portion; meanwhile, the zeaxanthin portion makes contact with Arm II and projects outside (Figures 9(b) and 9(c)). Tzeaxs present some observed hydrogen-bond interactions (Tyr334 and Ala333) and hydrophobic contacts (Cys275, Thr283, Leu321, and Val324) with fibrate derivatives, such as pemafibrate and ciprofibrate [50, 51].

In contrast to PPAR*α*, Tzeax does not bind to the Y-shaped ligand-binding pocket of PPAR*β*/*δ*. Instead, it is located outside of the LBD, on the H11 and H12 helices (Figure 9(d)), consistent with the previous finding that long-chain fatty acids (*C >* 20) do not fit into the pocket [52]. However, when a short fatty acid is added to Tzeax, the interaction happens as follows: the glucoside ester of Tzeax-13 and Tzeax-15 establishes contact with PPAR*β*/*δ* LBD through two hydrogen bonds at residues Ala342 and Glu295 (Tzeax-13; Figure 10(e)), and Thr292 and Ala342 (Tzeax-15; Figure 10(f)). In addition to hydrogen bonds, there are also hydrophobic contacts mainly with H3 (35%), H2′ (15%), and *β*-sheets (15%). Both Tzeax-13 and Tzeax-15 are in Arms II and III of the Y-shaped ligand-binding pocket of PPAR*β*/*δ* (Figures 9(e) and 9(f)). However, LBD of PPAR*β*/*δ* residues that make hydrogen bonds with Tzeax-13 and Tzeax-15 are different from those observed in eicosapentaenoic acid and GW2433; they make a network of hydrogen bonds via His323, His449, and Tyr473. Meanwhile, several residues (Arg284, Cys285, Leu339, and Val 348) interact with GW501515, a selective small-molecule [53], which are also observed in Tzeax-13 and Tzeax-15 interactions.

Figures 9(g) and 10(g) show how Tzeax interacts with PPAR*γ* LBD in the same way as it does with PPAR*α*, i.e., via hydrophobic contacts, with the exception that the interactions occur with H3 (55%), H4–H5 (18%), and H12 (9%). Tzeax can extend to Arms III, II, and X (Figure 9(g)). Meanwhile, the glucoside and fatty acid of the Tzeax-13 and Tzeax-15 are located in Arms II, III, and X of Y-shaped ligand-binding pocket, and zeaxanthin fraction surrounds LBD by H3, H4, and H12 (Figures 9(h) and 9(i)). In contrast to Tzeax, three hydrogen bonds were observed when the aliphatic chain increased from 7 to 12 carbons, namely, Tzeax13. Glucoside fraction of Tzeax-13 makes hydrogen bonds with residues Ile262 and Ser342 and the polar ring in the opposite site with residue Lys301 (H3; Figure 10(h)). A constant hydrophobic core interaction with H3 (43%) and H5 (17%) was also found. Similarly, the glucoside ester of Tzeax-15 establishes hydrogen bonds to H3 through Arg280 and loop H2'–H3 (Glu259 and Ile262) of PPAR*γ* LBD, establishing an interaction with Arm X (Figure 10(i)). Hydrophobic interactions with H3 (57%) and H5 (13%) are also present. These findings suggest that Tzeax-13 and Tzeax-15 could be partial agonists of PPAR*γ* because they share similar molecular interactions through some amino acid residues observed in partial agonists, such as amorfrutin, MLR-24, nTZDpa, and BVT.13 [54]. It has been recognized that there are different mechanisms of stabilization of LBD between full and partial agonists, explaining the differences in the grade of transactivation. Rosiglitazone and MRL-20, two full agonists, stabilize H3 and H12 helices of PPAR*γ* LBD through hydrogen bonds via Tyr473 and His449 [55]. Meanwhile, partial agonists, such as amorfrutin, MLR-24, nTZDpa, and BVT.13, tend to stabilize helix H3 and the *β*-sheet region, but at the same time destabilize helix H12. The binding to helix H3 and *β*-sheet through hydrogen bonds via Ser342 and Arg388 along with several hydrophobic contacts, including Ile341 (*β*-sheet) and Cys285 (H3), enables its stabilization [54].

HB-ATV-8 formulation has several advantages since it comprises nanomicellar structures around 10 nm in diameter, composed of membrane lipids of *T. aquaticus* and the peptide Helix-Y^12^. The small size of micelles is achieved because the peptide is able to modify the diameter of micelles (~100 nm) formed by pure lipids [56]. It has been shown that this kind of nanostructure is stable in terms of thermodynamics and kinetics [57, 58]. In addition, nanomicelles with amphipathic peptides are efficient delivery systems and promoting transport across membranes [59, 60]. Additionally, lipid–peptide complexes are convenient for maintaining structural stability and lesser degradation of peptides [61].

Finally, it is important to consider that a ligand can interact with more than one of the members of PPARs (dual agonist and pan agonist), but the affinity and the strength of activation depend on the molecular interactions established with each receptor. Each ligand promotes a unique conformational change, leading to differential pattern of coregulator recruitment, which in turn might cause gene selective effects [62]. Therefore, further understanding of the synergistic or antagonistic effects of HB-ATV-8 components as PPARs ligands is necessary to discard the possible disadvantage of administrating more than one ligand.

## 4. Conclusions

In the present study, we use blind molecular docking to show that the peptide Helix-Y^12^, Tzeax, Tzeax-13, and Tzeax-15, components of HB-ATV-8, favorably interact with the PPARs LBD. Although the interaction is mediated mainly by hydrophobic contacts, Helix-Y^12^, Tzeax-13, and Tzeax-15, share specific hydrogen bond interactions previously reported for known PPAR ligands. The Helix-Y^12^ fills the entire Y-shaped ligand-binding pocket of PPAR*α* and PPAR*γ*, but its contact with Arm III is partial. In the case of PPAR*β*/*δ*, there is a slight difference; the peptide establishes partial contact with Arm II. In contrast, Tzeaxs are constantly located in Arms II and III of the Y-shaped ligand-binding pockets of PPAR*α* and PPAR*γ*. Meanwhile, in PPAR*β*/*δ* they only occupy Arm II.

Previous study in our laboratory has demonstrated that the nasal administration of HB-ATV-8, containing Tzeaxs and Helix-Y^12^, reduces hypertriglyceridemia and vascular and hepatic lesions, including fibrosis, induced by a high-fat diet enriched with cholesterol in pigs and rabbits [29, 30]. In this line, PPAR*α* agonists are useful drugs for reducing serum triglycerides [63]. It is well known that PPAR*α* activated with fibrates and its derivatives reduce triglycerides-rich lipoproteins promoting fatty acids uptake and oxidation and increasing lipoprotein lipase activity [15, 64]. Preclinical [62, 65–67] and clinical [68–70] studies demonstrated that PPAR has an important role in NAFLD and non-alcoholic fatty steatohepatitis (NASH). PPAR*α* agonist counteracted dietary-induced NASH through PPAR*α* transrepression of signaling pathways that participate activator protein-1 and NF-*κ*B [66]. On the other hand, activation of PPAR*β*/*δ* with GW501516 leads to a reduction of hepatic fat accumulation and inflammation. In NAFLD/NASH animal models, elafibranor, a dual agonist PPAR*α*–*β*/*δ*, reduces hepatic steatosis, inflammation, and fibrosis [71, 72]. As well as patients with NASH, dyslipidemia, and prediabetic, elafibranor reduces plasma lipids and hepatic inflammation biomarkers [73, 74]. GFT505, a dual PPAR*α*–*β*/*δ*, demonstrated liver-protective effects on steatosis, inflammation, and fibrosis in animal models of NAFLD/NASH and liver fibrosis [75]. Therefore, activating more than one PPAR family member by a suitable exogenous ligand (dual- or pan-agonist) could represent an effective therapy against several metabolic alterations, such as hypertriglyceridemia, NAFLD, even fibrosis, and inflammation. Furthermore, according to our molecular docking results, the nanoparticle components, such as Helix-Y^12^ and Tzeax, might activate both PPAR*α* and PPAR*β*/*δ*, which would help us explain the protective effects against hypertriglyceridemia, vascular, and liver lesions.

## Figures and Tables

**Table 1:** Structural comparison of PPARs. Root mean square deviation [Å], protein fold similarity, and sequence identity [%]. All metrics evaluated with the TM-align tool [45].

PPAR	RMSD	TM-score	Seq id

*α–δ*	1.74	0.93	0.71
*α–γ*	1.48	0.97	0.64
*δ–γ*	1.02	0.96	0.66

**Table 2:** Main characteristics of Helix-Y^12^ interaction with PPARs.

	PPAR*α*	PPAR*β*/*δ*	PPAR*γ*

∆*G_b_*	−8.9	−7.9	−8.0
			
Interaction with Y-shaped ligand-binding pocket	Arm I, II, III*^a^*, and X	Arm I, II, III and X*^a^*	Arm I, II, III*^a^*, and X

			
Amino acid residues involved in H-bond (Receptor Helix-Y^12^)	Tyr314*^b^*–Cys1	Asn343–Leu8	Ser289*^b^*–Cys1
	Tyr464*^b^*–Cys1	Asn343–Gln9	Ile262–Val5
	Ala250–Ser10	Lys265–Ser10	Lys275–Leu8
		Arg284–Ser10 (2x)	Lys275–Gln9
			Ser464–Gln9

			
Hydrophobic contacts (mainly)	H3 (30%)	H3 (29%)	H3 (37%)
	H2′ (13.3%)	H5 (14%)	H5 (15%)
	H2′–H3 loop (13.3%)	H2′–H3 (11%)	H12 (11%)
	*β*2/*β*3 loop (10%)	H7 (11%)	H11–H12 (11%)

			
Advantages	Stablish two hydrogen bonds with residues of helix H12 important to LBD stabilization.	Hydrogen bonds are in Arm II and entrance.	Clamp surrounds H3, contacts with H12through a H-bond and several hydrophobic interactions, reaching loop H11–H12 from outside.
			

^a^Partial contact.

^b^Previously reported amino acids interacting with endogenous/exogenous ligands.

## Data Availability

The generated data of all systems studied here follow the FAIR principles and can be accessed and visualized at the MDdb Science Gateway at https://www.md-db.org with Study ID 690004.
